# Inherited aortopathy assessment in bicuspid aortic valve disease relative

**DOI:** 10.1186/1532-429X-18-S1-P341

**Published:** 2016-01-27

**Authors:** Malenka M Bissell, Luca Biasiolli, Abhishek Oswal, Margaret Loudon, Hugh Watkins, Stefan Neubauer, Saul G Myerson

**Affiliations:** grid.4991.50000000419368948Division of Cardiovascular Medicine, Radcliffe Department of Medicine, University of Oxford, Oxford, United Kingdom

## Background

Bicuspid aortic valve (BAV) is known to be heritable and often shows a concomitant aortopathy which was thought also to be present in some family members. While one recent study reported normal ascending aortic diameters in family members of the general BAV population, little is known about aortic function in such family members. We used advanced aortic cardiovascular magnetic resonance (CMR) and peripheral blood analysis to assess evidence for an underlying aortopathy in family members of bicuspid aortic valve patients.

## Methods

We prospectively enrolled 229 participants in total. This included 42 families (42 BAV index cases and 132 family members) in whom at least one member was affected by BAV. Participants over 6 years of age underwent CMR, and those under 6 years echocardiography. We also recruited 55 age and sex-matched healthy volunteers. Advanced aortic assessment included aortic diameters, pulse wave velocity (PWV), arterial stiffness, maximum rate of systolic dysfunction (MRSD) and distensibility by CMR, total peripheral resistance (TPR) by Vicorder^©^ as well as circulating matrix metalloproteinases (MMP) 2 and 9 from a peripheral blood sample. A subset of 10 family members also underwent comprehensive 4D flow MRI assessment.

## Results

11% (14/132) family members were found to have a BAV. The remaining 118 family members with a normal functioning tricuspid aortic valve had an average age of 38.7 years with a mean pulse pressure 57 mmHg. Compared to sex, age and blood-pressure matched healthy volunteers all family members had normal sinus, ascending and descending aortic diameters. There was no difference in PWV (arch 5.9 vs 5.0 m/s, p = 0.31; descending aorta 8.5 vs 8.7 m/s, p = 0.85), ascending, proximal and distal descending aortic strain (ascending aorta 0.16 vs 0.19; p = 0.41). MRSD (ascending aorta 0.23 vs 0.22%/ms, p = 0.61) and distensibility (ascending aorta 4.0 vs 4.4 1/mmHg, p = 0.65), TPR (1.03 vs 1.06pru, p = 0.56), MMP2 and MMP9. In the subset of family members undergoing advanced 4D flow MRI assessment, there was no difference in flow angle (8.75 vs 7.75°, p = 0.53), flow displacement (2.73 vs 2.44 mm, p = 0.67) and wall shear stress compared to healthy volunteers (0.69 vs0.59 N/m^2^, p = 0.22).

## Conclusions

Family members with (normal) trileaflet aortic valves of patients with a bicuspid aortic valve have normal aortic size and function. These findings point towards the importance of haemodynamic factors rather than additional haemodynamic-independent mechanisms in the aortopathy of bicuspid aortic valve patients.

